# SEOM guidelines for the management of Malignant Melanoma 2015

**DOI:** 10.1007/s12094-015-1450-4

**Published:** 2015-12-15

**Authors:** A. Berrocal, A. Arance, E. Espinosa, A. G. Castaño, M. G. Cao, J. L. G. Larriba, J. A. L. Martín, I. Márquez, A. Soria, S. M. Algarra

**Affiliations:** Servicio de Oncología Médica, Consorcio Hospital General Universitario de Valencia, Avda. Tres Cruces 2, 46014 Valencia, Spain; Hospital Clinic I Provincial de Barcelona, Barcelona, Spain; Hospital Universitario la Paz, Madrid, Spain; Hospital Universitario Marqués de Valdecilla, Santander, Spain; Hospital Universitario Quirón Dexeus, Barcelona, Spain; Hospital Universitario Clínico San Carlos, Madrid, Spain; Hospital Universitario 12 de Octubre, Madrid, Spain; Hospital General Universitario Gregorio Marañón, Madrid, Spain; Hospital Universitario Ramón y Cajal, Madrid, Spain; Clínica Universitaria de Navarra, Pamplona, Spain

**Keywords:** Melanoma, Metastatic, Adjuvant, Immunotherapy, b-raf

## Abstract

All melanoma patients must be confirmed histologically and resected according to Breslow. Sentinel node biopsy must be done when tumor is over 1 mm or if less with high-risk factors. Adjuvant therapy with interferon must be offered for patients with high-risk melanoma and in selected cases radiotherapy can be added. Metastatic melanoma treatment is guided by mutational BRAF status. BRAF wild type patients must receive anti-PD1 therapy and BRAF mutated patients BRAF/MEK inhibitors or anti-PD1 therapy. Up to 10 years follow up is recommended for melanoma patients with dermatologic examinations and physical exams.

## Methodology

As most of the knowledge on the treatment for this disease has come in the last 5 years and from phase III clinical trials for the development of this guideline, the authors have reviewed all phase III trials regarding the main aspects of this guideline and also the main guidelines on this disease. Recommendation and evidence have been graded according to the guidelines development recommendations [1].

## Surgical management of melanoma

When a confident clinical diagnosis of melanoma is made excision biopsy should be performed. Excision biopsy must have 2 mm (no larger, in order to not alter the lymphatic drainage) of lateral surgical margins and deep margins in the subcutis (*Grade recommendation A*; *Level of Evidence 1a*).

Regarding the surgical margins for melanoma in situ or lentigo maligna complete excision should be made. There are no randomized controlled trials about margins. A surgical margin of 5–10 mm has shown low recurrences rates (*Grade recommendation A*; *Level of Evidence 2c*). When melanoma diagnostic is confirmed, the excision should be performed with deep margins extending to the fascia (*Grade recommendation B*; *Level of Evidence 2b*). Surgical margins according to the Breslow thickness has been tested in seven randomized trials (*Grade recommendation A*; *Level of Evidence 1a*). Present data show surgical margins of 1 cm for melanomas up to 1 mm Breslow. For 1–2 mm of Breslow, margins could be of 1–2 cm (there have been studies comparing 1–3 and 2–5 cm). Above 2 mm of Breslow, 2 cm is recommended. A randomized study demonstrated that 1 cm of margin increased the locoregional recurrence and also risk of melanoma specific death over 3 cm of margin (relative rate of melanoma death was estimated to be 24 % higher in the 1 cm group than the 3 cm group on univariate analysis [hazard ratio (HR) 1.24; 95 % confidence interval (CI) 1.00–1.52; *p* = 0.05] [2]. Studies comparing 2 vs 4 cm did not reveal any benefit for wider margins in melanomas of more than 2 mm of Breslow. Only limited data are available for melanomas of more than 4 mm of thickness. A surgical margin of more than 3 cm is not beneficial.

Some patients are considered for Sentinel node biopsy. In melanomas of more than 1 mm of Breslow a sentinel node biopsy must be performed (*Grade recommendation A*; *Level of Evidence 1a*). In patients with melanomas of 0.75–1 mm of Breslow and any risk factor as ulceration, less than 40 years old, Clark level IV, regression or increased mitotic rate, a sentinel node biopsy must be considered as some studies have demonstrated sentinel node metastases up to 20 % of cases [3] (*Grade recommendation B*; *Level of Evidence 1a*). Complete lymph node dissection consists of anatomically thorough dissection of the involved nodal basin. It must be performed if sentinel node is positive or there are clinically positive nodes (stages IIB or IIIC). (*Grade recommendation A*; *Level of Evidence 2a*).

In case of metastatic disease, surgical therapy of metastases may be indicated. Data from retrospective studies demonstrated survival rates of 20–30 % at 5 years after surgical removal of single metastases (*Grade recommendation B*; *Level of Evidence 2b*).

### Adjuvant therapy

Despite surgical treatment, an important part of the patients may relapse. Some of the most important prognostic factors for relapse are the tumoral thickness, the mitotic rate, the presence of ulceration, and lymph node involvement. Depending on its presence or absence, disease can be divided into:

- Low-risk disease: stages I and IIA (T1–3 without ulceration and T1–2 with ulceration). There is no evidence of efficacy with adjuvant treatment in this context.
- High-risk disease: stages IIB-C (T4 or with ulceration) and stage III (N positive).

High-risk patients are considered candidates for adjuvant treatment. The first drug approved in the high-risk disease context was Interferon alpha (IFNa), based on the results of the ECOG 1684 trial, where the high-dose scheme (induction treatment with IFNa 20 MU/m^2^ iv × 5 days/week × 4 weeks, followed by maintenance treatment with IFNa 10 MU/m^2^ sc × 3 days/week × 11 months) demonstrated a significant benefit in relapse-free survival versus observation [4]. Although initially this benefit extended to overall survival, a follow-up superior to 12 years showed no significant differences [5]. After that, many studies have evaluated the efficacy and the toxicity profile of this drug relative to other agents or different schemes and dosage.

Alternative schemes with low-dose IFNa for 18 months showed a significant improvement in RFS for stages II, but not significant in overall survival [6]. However, evaluated in the global context of high-risk population (stages II and III), not only showed its inferiority versus the high-dose scheme in relapse free survival, but also its inferiority with the observational arm [7]. The poor results with low-dose schemes could not be improved by the addition of initial induction schemes neither with longer treatments.

With all of these conflicting results about benefit in OS, recently several meta-analyses have tried to answer this question. Whereas one of them confirmed the significant improvement in RFS, but not for OS [8], two other meta-analyses have demonstrated a significant benefit in OS [9, 10]. Nevertheless, none of them have been able to respond the answer about the optimal IFNa treatment scheme and which subgroup of patients will be the best candidates to receive it.

Given these results, high-risk melanoma patients should receive interferon adjuvant therapy (*Grade recommendation A*; *Level of Evidence 1a*).

It is expected a change in the therapeutic scene in next years with the publication of the results of trials evaluating new immunotherapy agents and BRAF/MEK inhibitors.

## Radiotherapy

Adjuvant radiotherapy is rarely necessary for excised local melanoma and should be considered in case of inadequate resection margins of lentigo maligno, desmoplastic neurotropic melanoma, which tends to be locally aggressive o R1 resections of melanoma metastases when surgery is not adequate (*Grade of recommendation B*; *level of evidence 2b*).

Adjuvant radiotherapy may be considered for select patients with clinically positive nodes and features predicting a high risk of nodal basin relapse. A prospective randomized trial with 250 non-metastatic patients and radiotherapy (48 Gy) versus observation did not demonstrate benefit in overall survival in the patients treated but it demonstrated improvement in tumor control at lymph node region [11]. Adjuvant radiotherapy is recommended when there are three affected lymph nodes, or there is capsule penetration or there is a lymph node metastases greater than 3 cm (*Grade of recommendation C*; *level of evidence 1b*). In addition, postoperative radiotherapy should be used after resection of a lymphatic recurrence (*Grade of Recommendation C*, *level of evidence 2b*).

Many different options are available to patients presenting with stage III in-transit metastases. Treatment is based on the size, location and number of lesions. Radiotherapy can be an alternative when these lesions are inoperable or others alternatives as isolated limb perfusion is not a possibility (*Grade of recommendation C*; *level of evidence 4*).

## Locoregional metastases

Patients with satellite and in-transit metastases should be treated within the context of clinical studies if possible.

Surgical therapy of in-transit metastases shall be performed when—with lack of indications of distant metastasis—there is a possibility of macroscopic and microscopic complete removal of the metastases (*Grade of recommendation B*, *level of evidence 4*).

In the presence of multiple, inoperable, locoregional cutaneous metastases on an extremity, regional chemotherapy as isolated limb perfusion comes into consideration (*Grade of recommendation B*, *level of evidence 4*). Metastases outside of the perfusion area may be treated by intratumoral injection of interleukin-2 (*Grade of recommendation B*, *level of evidence 4*). Other procedures such as radiation therapy, electrochemotherapy, cryosurgery, or laser destruction may also be applied for local tumor control (*Grade of recommendation C*, *level of evidence 4*). Fundamental superiority of one over the other has not been proven and their use depends on individuals factors.

## Treatment of metastatic disease

The presence of BRAF-V600 mutation is mandatory for the use of BRAF and MEK inhibitors (*Grade of recommendation A*, *level of evidence 1a*). Validated BRAF-V600E/K test methods are tissue based and provide qualitative data (positive or negative).

### First line therapy in BRAF-mutant, advanced (IIIC/IV) melanoma

Three phase III trials have demonstrated that the combination of a BRAF inhibitor and a MEK inhibitor is more active than single agent BRAF inhibitor: *COMBI*-*D* dabrafenib and trametinib vs dabrafenib [12]; *COMBI*-*V* dabrafenib and trametinib vs vemurafenib [13]; and *COBRIM* vemurafenib and cobimetinib vs vemurafenib [14]. (Over two-thirds of patients achieve an objective response, time to progression lies in the range of 10–11 months and median overall survival is around 25 months) (*Grade of recommendation A*, *level of evidence 1a*.) A third combination (encorafenib and binimetinib) has shown activity in a phase Ib/II study and a phase III study is ongoing (NCT01909453).

Single agent BRAF inhibitors vemurafenib or dabrafenib must be considered (response rate 50 %, time to progression 6–8 months, median overall survival 16–18 months) if the combination with their companion MEK inhibitor cannot be used [15, 16] (*Grade of recommendation A*, *level of evidence 1a*). The MEK inhibitor trametinib as single agents is more active that DTIC, but less effective than BRAF inhibitors.

BRAF inhibitors are also active in patients with brain metastases response rate 30–39 %, overall survival 8 months [17]. Concomitant use with radiotherapy is not recommended due to the risk of increased toxicity (*Grade of recommendation A*, *level of evidence 2a*). The combination of BRAF and MEK inhibitors is currently being tested in this setting.

Anti-CTLA4 and anti-PD1 antibodies have not been directly compared with targeted therapies in advanced melanoma, but clinical trials have consistently demonstrated that its activity is not affected by BRAF mutational status [18] (*Grade of recommendation A*, *level of evidence 1a*).

Ipilimumab induces a significantly lower response rate than BRAF inhibitors, but approximately 20 % of patients achieve long-term survival (*Grade of recommendation A*, *level of evidence 1b*), and might be a reasonable option in patients with good performance status, low tumour burden and normal LDH level [18].

Nivolumab and pembrolizumab, as well as the combination of nivolumab and ipilimumab are active and have a higher response rate 43 and 33 % , respectively [19–21] (*Grade of recommendation A*, *level of evidence 1a*); however, long-term results and the optimal duration of therapy with these agents are not yet known.

Chemotherapy should only be considered as first line if the more active options are not available or contraindicated (*Grade of recommendation A*, *level of evidence 1a*).

### Second line therapy in BRAF-mutant advanced melanoma

The activity of BRAF and MEK inhibitors after immunotherapy has not been prospectively studied, but seems to be similar to that obtained in first line (*Grade of recommendation A*, *level of evidence 2b*).

Retrospective data about the efficacy of ipilimumab after BRAF inhibitors vary among investigators (median overall survival 5–12 months), but this result has been related to treatment adherence, poor performance status and rapid clinical deterioration.

The anti-PD1 antibodies nivolumab and pembrolizumab are superior to chemotherapy in term of response rate (26–31 %) and time to progression (3.7–4.7 months) in patients pretreated with ipilimumab or BRAF inhibitors (*Grade of recommendation A*, *level of evidence 1b*).

### Treatment of BRAF wild type, advanced (IIIC/IV) melanoma

Several chemotherapeutic agents (dacarbazine, temozolamide, fotemustine, carboplatin, cisplatin, and paclitaxel among others) have been tested in randomized clinical trials with similar response rates (5–12 %) and suvival (<5 %) results (*Grade of recommendation A*, *level of evidence 1a*).

The results of two randomized phase III studies have led to the approval of Ipilimumab (3 mg/kg) for the treatment of metastatic melanoma in first and second-line setting, due to a significant improvement in median overall survival (10 months) and long-term survival (20 %) (*Grade of recommendation A*, *level of evidence 1a*). Response to ipilimumab may delay 12 weeks or more after treatment initiation and tumor response must be assessed after the completion of four doses. Immune adverse events (skin, gastrointestinal, liver, endocrine), usually resolve after steroid treatment or hormonal replacement [22, 23].

Nivolumab and pembrolizumab [19, 21], as well as nivolumab and ipilimumab [20], have a higher response rate and survival than Ipilimumab (*Grade of recommendation A*, *level of evidence 1a*) and have been approved by FDA and EMA (only single agent nivolumab and pembrolizumab). The optimal duration of therapy and long-term results with these agents is not yet known.

Results from case reports and a phase II study of KIT inhibitor imatinib suggest that some patients with KIT mutations (more common in acral lentiginous and mucosal melanomas) may respond (10–20 %) to KIT kinase inhibitor therapy (*Grade of recommendation C*, *level of evidence 2b*), but these agents have not been approved for this indication KIT as a therapeutic target in metastatic melanoma [24].

## Follow up

The objective of follow-up is the early detection of recurrences and secondary melanomas. The optimal duration of follow-up remains controversial (Table 1). Studies in stage I–III showed that 47 % of recurrences occurred within the first year after diagnosis and 32 and 80 % within the second and third years, respectively, and thorough follow-up is advocated for this time period. Late recurrences are well documented but only 5 % of recurrences occur after 10 years. Thus, a 10-year follow-up appears to be reasonable (*Grade of recommendation B*; *level of evidence 1b*). Patients with a primary melanoma are at increased risk for developing a second primary melanoma. Estimates of that increased risk range from 8 to 10 %. Although the most secondary melanomas occur within the first 2 years after the primary diagnosis of melanoma may even occur more than 30 years after, suggesting a need for life-long, regular dermatologic examinations (*Grade of recommendation B*; *level of evidence 3b*). Self-examinations by the patient are an essential component of follow-up and can lead to early recognition of recurrences of new melanomas. The patients should receive instructions on self-examination to detect a new melanoma or recognize a recurrence themselves (*Grade of recommendation B*; *level of evidence 3b*).

**Table 1:** Recommendations table

Surgery		
All melanoma must be biopsied	A	1a
Surgical margins should be Breslow adapted	A	1a
Melanomas of more than 1 mm should undergo sentinel node biopsy	A	1a
Melanomas of 0.75 mm should undergo sentinel node biopsy if there are risk factors	B	1a
Lymph node resection should be performed if sentinel node is positive or clinically evident	A	2a
Solitary metastases must be surgically removed	B	2b
Adjuvant therapy		
High risk melanoma patients should receive interferon adjuvant therapy	A	1a
If surgical margins are affected adjuvant radiotherapy may be added	B	2b
Adjuvant radiotherapy should be considered if more than 3 nodes are present, one is larger than 3 cm or capsule is broken	C	1b
Locoregional disease		
Palliative radiotherapy can be used in transit metastases	C	4
Surgery can be used for in transit metastases	C	4
Isolated limb perfusion can be used for in transit metastases	C	4
Metastatic disease		
B-RAF determination should be done for all metastatic patients	A	1a
Combined B-RAF/MEK inhibition should be offered for BRAF mutated patients	A	1a
Single agent BRAF inhibitor is appropriate is there is contraindication for MEK inhibitor	A	1a
BRAF inhibitors may be used in brain metastases	A	2a
Immunotherapy results are not affected by BRAF status	A	1a
Anti PD1 therapy is an alternative for BRAF mutated patients whose disease is not aggressively progressing	A	1a
Chemotherapy is an option if no other therapy could be available	A	1A
Patients treated with immunotherapy must be offered BRAF/MEK therapy as second line	A	2b
Patients treated with BRAF/MEK inhibitors must be offered anti-PD1 therapy	A	2a
Anti-PD1 therapy is the first option for BRAF wild type patients	A	1a
Chemotherapy may be used as second line for BRAF wild type patients	A	1a
KIT mutated melanomas may be offered KIT kinase inhibitors	C	2b
Follow up		
Ten year follow up must be offered	B	1b
Lifelong skin examination is recommended	B	3b
Self-examination is recommended	B	3b
Physical examination is recommended	A	2b
Lymph node sonogram is recommended if physical exam is not clear	A	1A

Physical examinations in stage I–III disease have proven to be the most effective procedure for early recurrence detection [25] and shall be performed in all melanoma patients during follow-up (*Grade of recommendation A*; *level of evidence 2b*).

Routine blood testing to detect recurrence is not recommended (*Grade of recommendation D*; *level of evidence 4*). Early detection of locoregional lymph node metastases is of particular significance. In a meta-analysis of 74 trials, lymph node sonography proved to be the most sensitive and most specific procedure for the detection of locoregional lymph node metastases and is the particular interest in patients with and equivocal lymph node physical exam, patients without sentinel lymph node biopsy (SLNB) or patients with a positive SLNB who did not undergo complete lymph node disection (*Grade of recommendation A*; *level of evidence 1a*).

Overall, a general recommendation about imaging procedure is not possible, because there are no studies assessing how the early detection of a recurrence could have an impact in the overall survival with the new treatments, as immunotherapy. In view of the current data, it is possible that an early detection of recurrence could have an impact in the response and evolution with the new treatments. Individual follow-up exams may be conducted in a risk-adapted fashion, trimonthly intervals in high risk of recurrence and in patients with decreasing risk, follow-up intervals may be extended from 6 to 12-months.
